# Genomic Investigation of Desert *Streptomyces huasconensis* D23 Reveals Its Environmental Adaptability and Antimicrobial Activity

**DOI:** 10.3390/microorganisms10122408

**Published:** 2022-12-05

**Authors:** Ying Wen, Gaosen Zhang, Ali Bahadur, Yeteng Xu, Yang Liu, Mao Tian, Wei Ding, Tuo Chen, Wei Zhang, Guangxiu Liu

**Affiliations:** 1 Key Laboratory of Desert and Desertification, Northwest Institute of Eco-Environment and Resources, Chinese Academy of Sciences, Lanzhou 100864, Gansu, China; 2 Key Laboratory of Extreme Environmental Microbial Resources and Engineering, Lanzhou 100864, Gansu, China; 3 University of Chinese Academy of Sciences, No.19A Yuquan Road, Beijing 100049, China; 4 State Key Laboratory of Cryospheric Sciences, Northwest Institute of Eco-Environment and Resources, Chinese Academy of Sciences, Lanzhou 100864, Gansu, China; 5 State Key Laboratory of Microbial Metabolism, School of Life Sciences & Biotechnology, Shanghai Jiao Tong University, Shanghai 200030, China

**Keywords:** *Streptomyces* genome, bioactive, biosynthetic gene clusters, ecological adaptations

## Abstract

The harsh climatic conditions of deserts may lead to unique adaptations of microbes, which could serve as potential sources of new metabolites to cope with environmental stresses. However, the mechanisms governing the environmental adaptability and antimicrobial activity of desert *Streptomyces* remain inadequate, especially in extreme temperature differences, drought conditions, and strong radiation. Here, we isolated a *Streptomyces* strain from rocks in the Kumtagh Desert in Northwest China and tested its antibacterial activity, resistance to UV-C irradiation, and tolerance to hydrogen peroxide (H_2_O_2_). The whole-genome sequencing was carried out to study the mechanisms underlying physiological characteristics and ecological adaptation from a genomic perspective. This strain has a growth inhibitory effect against a variety of indicator bacteria, and the highest antibacterial activity recorded was against *Bacillus cereus*. Moreover, strain D23 can withstand UV-C irradiation up to 100 J/m^2^ (D10 = 80 J/m^2^) and tolerate stress up to 70 mM H_2_O_2_. The genome prediction of strain D23 revealed the mechanisms associated with its adaptation to extreme environmental and stressful conditions. In total, 33 biosynthetic gene clusters (BGCs) were predicted based on anti-SMASH. Gene annotation found that *S. huasconensis* D23 contains several genes and proteins associated with the biosynthesis of factors required to cope with environmental stress of temperature, UV radiation, and osmotic pressure. The results of this study provide information about the genome and BGCs of the strain *S. huasconensis* D23. The experimental results combined with the genome sequencing data show that antimicrobial activity and stress resistance of *S. huasconensis* D23 was due to the rich and diverse secondary metabolite production capacity and the induction of stress-responsive genes. The environmental adaptability and antimicrobial activity information presented here will be valuable for subsequent work regarding the isolation of bioactive compounds and provide insight into the ecological adaptation mechanism of microbes to extreme desert environments.

## 1. Introduction

Despite tremendous advances in human medicine, infectious diseases caused by bacteria, fungi, and viruses remain a major threat to public health [1]. Meanwhile, antibiotic resistance has led to a global health crisis, with incurable infections [2], and discovering novel effective antimicrobial compounds is significantly important [3]. Currently, natural products from microorganisms remain the main source of novel antimicrobial drugs, with more than 50% of drugs obtained from natural products [4,5].

Among Actinomycetes, *Streptomyces* is considered to be the richest source of bioactive secondary metabolites [6]. Since the first isolation of streptomycin from bacteria by *Streptomyces griseus* [7] in 1944, a large number of unique and potent antimicrobial and antitumor products encoded by *Streptomyces* have gained widespread scientific attention [8]. *Streptomyces* can produce a variety of bioactive secondary metabolites that are antagonistic to plant and human pathogens, and many also have the ability to promote plant growth [9,10,11]. Currently, most of the *Streptomyces* isolated from traditional habitats duplicate previous isolates, which is costly and inefficient. Therefore, an increasing number of researchers are exploring the isolation of new *Streptomyces* resources from extreme environments or unexplored habitats on the earth [12]. Arid and semi-arid ecosystems occupy almost one-third of the Earth’s terrestrial surface, of which more than 95% are desert ecosystems with extreme aridity, lack of nutrients, extreme temperature differences, and superb UV irradiation [13,14]. Nutrient-poor microbial communities in arid ecosystems contain more genes not found in public reference databases, and oligotrophic microbial communities are a rich source of new functions [15]. In addition, desert environments have been found to promote the unique evolution of *Streptomyces* biosynthetic potential [16].

*S. huasconensis* [17] was first identified in a Chile salt lake in the Atacama Desert region. Subsequently, researchers isolated the smallest lasso peptide from *S. huasconensis* HST28^T^, Gly1-Asp7 Macrocycle [18]. This reveals that *S. huasconensis* HST28^T^ has the potential to produce new metabolites. Despite the progress made in secondary metabolite isolation for this strain, understanding the genomic ability of this strain to encode secondary metabolites and the ecological adaptation mechanisms is an important limitation to our further development of the biochemistry of this strain, especially the molecular isolation of compounds with applications.

In this study, we sequenced the whole genome of *S. huasconensis* D23 isolated from Kumtagh Desert rocks and investigated the effect of its crude ferment on the growth of indicated bacterial strains, such as *Staphylococcus aureus* (*S.*a), *Micrococcus luteus* (*M.*l), *Bacillus thuringiensis* (*B.*th), *Pseudomonas putida* (*P.*p), *Lactococcus lactis* (*L.*l), *Bacillus cereus* (*B*.c), *Escherichia coli* (*E.*c), and *Staphylococcus epidermidis* (*S.*ep). In addition, we explored the resistance of *S. huasconensis* D23 to UV-C and H_2_O_2_. Moreover, we compared *S. huasconensis* D23 with three strains of bacteria with high similarity to broaden the genomic basis for functional and comparative analyses with the focus on the genetic basis of microbial adaptation to the environment. In addition, the genome sequences of some type strains of *Streptomyces* species published in the GenBank database were used to determine evolutionary relationships and analyze pan-genome characteristics. The outcome of the current study provides information on genome sequence and secondary metabolite biosynthetic gene clusters (smBGCs), which is valuable for researchers interested in the isolation of bioactive secondary metabolites from Actinomycetes.

## 2. Materials and Methods

### 2.1. Sampling and Bacterial Strain Isolation

Bacterial strain D23 was isolated from rocks in the Kumtagh Desert, Northwest China. We used sterile cotton swabs dipped in sterilized saline to wash off the dust attached to the rock, and the soil suspension was diluted to 10^−5^ before being spread onto R2A agar [19]. The isolation plate was incubated aerobically at 25 °C for 7 days and purified using the streaking method. The axenic culture was preserved in glycerol stock solutions (20%, *v*/*v*) at −80 °C. Uploading the 16S rRNA gene sequence of strain D23 to the EZ Taxon database (EzBioCloud) [20] showed that the recovered bacterium belonged to the genus *Streptomyces* spp.

### 2.2. Screening of the Antibacterial Activity

(1) The spores of strain D23 were inoculated in Tryptic Soy Broth medium (Tryptone 17.0 g/L, Peptone 3.0 g/L, Sodium chloride 5.0 g/L, Dipotassium hydrogen phosphate 2.5 g/L, Glucose 2.5 g/L, pH 7.3 ± 0.2) at 28 °C, 200 rpm for 48 h for activation. Then, the spore solution was transferred to a liquid fermentation medium according to 10% inoculum and incubated at 30 °C, 200 rpm for 168 h; the fermented medium was collected and centrifuged at 7000 rcf/min for 10 min to obtain the fermentation supernatant. Three parallel fermentation broths were extracted with ethyl acetate at a volume ratio of 1:1 for 12 h. The extracted upper organic phase was removed by rotary evaporator, dissolved with 1 mL of methanol, and stored at −20 °C until the determination of bacterial inhibitory activity.

(2) Indicator strains plate decantation: Lysogeny broth medium (Tryptone 10 g, Yeast extract 5 g, NaCl 10 g, Agar 20 g, Distilled water 1000 mL; Adjust pH to 7.0) was autoclaved at 121 °C for 20 min, cooled to 55 °C, and added to indicator strain growth solution. It was then mixed, poured into the plate, and cooled until the agar medium solidified. We punched out 6 mm round holes with a sterile hole puncher, collected 20 µL of methanol solubilized extract, and added it into the holes, using methanol as the negative control and kanamycin (2 mg/mL) as the positive control. In the next step, we added the samples to the holes in the agar medium and incubated it overnight at 37 °C in a constant temperature with the incubator upside down. The size of the inhibition circle was measured using the crossover method.

### 2.3. Screening UV-C Radiation-Resistance and H_2_O_2_ Tolerance of the Strain

Fresh *Streptomyces* spores were scraped onto R2A plates to produce spore suspension, and the cell suspension was adjusted to OD 600 = 1. The UV-C irradiation method was referenced by Liu [21], and each irradiation intensity’ sample was divided into 3 aliquots. One of them did not receive radiation, while the other two aliquots were exposed to UV-C irradiation with doses of 20, 40, 60, and 100 J/m^2^, respectively. After irradiation, the spore solution was diluted to 10^3^–10^4^ folds and plated on R2A agar medium. *Escherichia coli* DSM 30,083 was used as the negative control. The plates were incubated at 28 °C for 3~7 days and the colonies were counted.

The spores were inoculated into R2A medium and cultured for 3 days. Cells in the logarithmic growth phase were washed twice and resuspended in 0.9% NaCl. After the addition of different concentrations of H_2_O_2_ and treatment for 120 min, cells were centrifuged and washed, coated on solid R2A plates, and placed in an incubator at 28 °C. Three replicates were set up for each oxidant concentration and counted after they grew as colonies.

### 2.4. Whole-Genome Sequencing and Annotation Analysis

Strain D23 was incubated in TSB medium at 28 °C for 3 days. Bacterial genomic DNA was extracted using a bacterial genomic DNA extraction kit according to the manufacturer’s instructions (Omega Bio-tek, Inc., Norcross, GA, USA). Sequencing was performed using the three-generation single-molecule real-time sequencing method, and the experimental procedure was performed according to the standard protocol provided by Oxford Nanopore Technologies (Oxford, UK), including sample quality testing, library construction, and library quality testing. Library Gene assembly was performed using Flye-2.8.2. Genome annotation, and gene prediction were performed using RAST [22] and the NCBI Prokaryotic Genome Annotation Pipeline (PGAP) [23,24,25]. A database of species most closely related to *S. huasconensis* D23 was established using TYGS (https://tygs.dsmz.de/user_reque sts/new, accessed on 3 December 2022) to build a genome-based phylogenetic tree. The identification of secondary metabolite BGCs was performed by Antibiotics and Secondary Metabolites Analysis Shell (anti-SMASH) [26]. In this study, the genomic data of *S. huasconensis* D23 was uploaded to anti-SMASH 6.0.1 (https://antismash.secondarymetabolites.org, accessed on 3 December 2022) for analysis using default parameters. Gene functions were analyzed by BLASTP using Cluster of Orthologous Groups (COG) and KEGG on the WebMGA server [27]. The closely related species *S. alfalfae* XY25, *S. alboniger* ATCC12461, and *S. kanamyceticus* ATCC12853 were obtained from the NCBI database and compared with strain D23. Next, the protein sequences of four strains were uploaded for comparison, and the annotation of directly homologous gene clusters to OrthoVenn2 (https://orthovenn2.bioinfotoolkits.net/home, accessed on 3 December 2022) [28] was performed. The BPGA pipeline was used to perform model extrapolations of the *S. huasconensis* and pan-genome/core genome by applying default parameters [29].

## 3. Results

### 3.1. Taxonomic Studies of Streptomyces sp. D23

**Figure 1:**
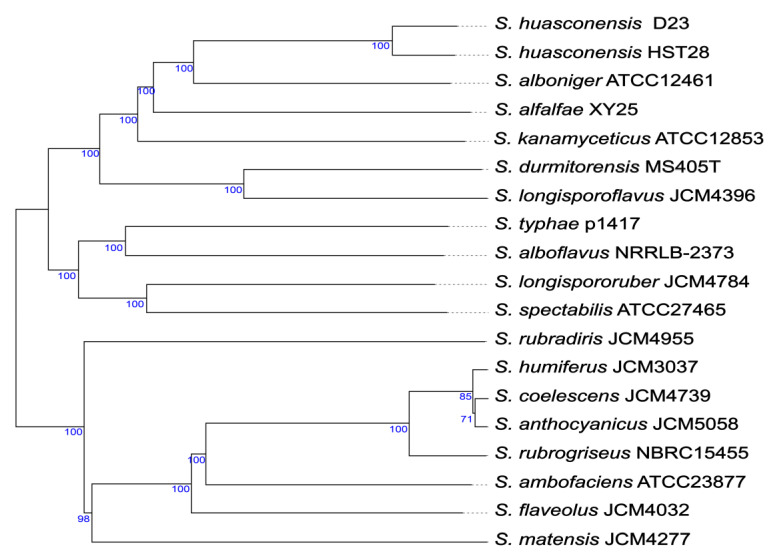
Phylogenomic tree based on genome sequences in the TYGS tree inferred with FastME 2.1.6.1 [30] from Genome BLAST Distance Phylogeny approach (GBDP); distances calculated from genome sequences. The branch lengths are scaled in terms of GBDP distance formula d5. The branches above are GBDP pseudo-bootstrap support values >60% from 100 replications, with an average branch support of 94.5%.

A genome-wide phylogenetic tree was constructed to determine the evolutionary relationships of strain D23 with other *Streptomyces* (Figure 1). Among the strains with published genome sequences, strain D23 had the closest evolutionary distance to the salt-tolerant actinomycete *S. huasconensis*, which was isolated from a high-altitude saline wetland at the Chilean Altiplano; after the comparison of the ANI value, we identified that strain D23 belongs to *S. huasconensis*.

### 3.2. Screening for the Potential Activity

Strain D23 showed inhibition against all used microorganisms. The results of bacterial inhibition on different fermentation media under the same growth conditions were significantly different (Table 1). We measured the transparent (inhibited) areas on the agar plates as an indicator of the efficacy of the antimicrobial compounds, using the zone diameter of the inhibited area (mm) to indicate the antimicrobial activity. The highest antibacterial activity recorded was against *Bacillus cereus* with an inhibition diameter of 30 mm. The fermentation product in Gauze’s Synthetic Medium NO. 1 had a good inhibition effect on five of the selected seven indicator strains.

### 3.3. Survival Rates after Exposure to UV-C and H_2_O_2_ Tolerance

**Figure 2:**
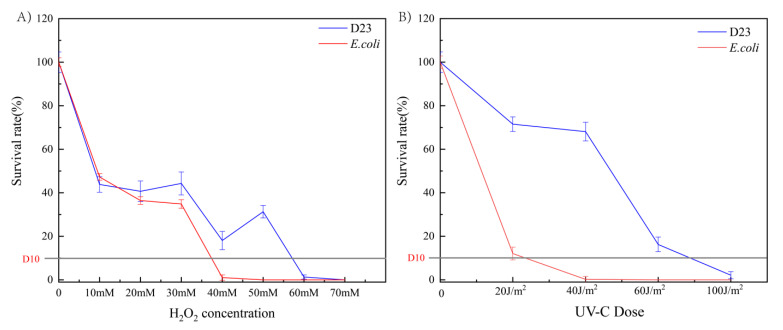
Survival rates of strain D23 under UV-C and hydrogen peroxide stress. (**A**) Survival rate of strain D23 and *E. coli* after exposure to 0–70 mM hydrogen peroxide stress; (**B**) survival rate of strain D23 and *E. coli* after exposure to UV-C radiation in a dose range of 0–100 J/m^2^. D10 values: dose required to kill 90% of cells.

To the resistance to UV-C radiation and antioxidant capacity of strain D23, we simulated the survival tests of the strain in the laboratory under UV-C irradiation and oxidation, and the results are shown in Figure 2. We found that the survival rate of strain D23 was much higher than that of the *E. coli* strain. When the concentration of hydrogen peroxide was 40 mM, the survival rate of strain D23 was 18.07%, while the survival rate of *E. coli* tended to be close to 0 (Figure 2A). The difference was even more apparent under UV-C irradiation, where the survival rate of strain D23 was 71.5% at an irradiation dose of 20 J/m^2^. In sharp contrast, the survival rate of *E. coli* plummeted to 12.09%. Eventually, at an irradiation dose of 80 J/m^2^, the survival rate of strain D23 dropped to about 10%, while *E. coli* was completely dead at 60 J/m^2^ (Figure 2B). Therefore, we suggest that the UV-C resistance and oxidative resistance that distinguish strain D23 from common strains are important physiological bases that allow strain D23 to survive in the harsh desert environment.

### 3.4. General Genome Features of S. huasconensis D23

**Figure 3:**
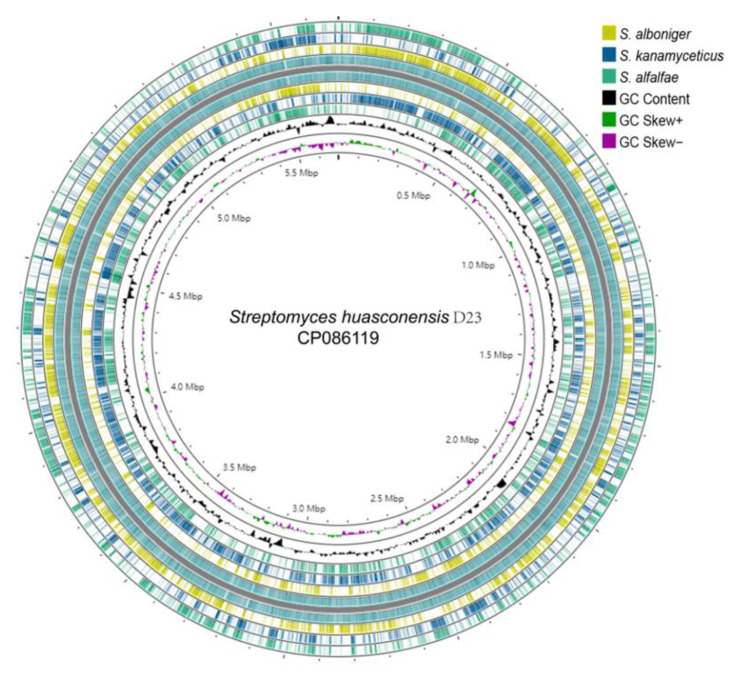
Circos showed that strain D23 contains (from outer to inner rings), contigs, coding sequences (CDs) on the forward strand, coding sequences (CDS) on the reverse strand, coding sequences of *S. alfalfae* XY25, *S. kanamyceticus* ATCC12853 and *S. alboniger* ATCC12461, GC skew, and GC content.

In total, 823,658,200 bases and 2,324,330 read counts with 300 bp read length were obtained as the raw sequence reads. The Flye-2.8.2 assembler was employed, resulting in the generation of a total of 1 contigs and 8.2 Mb assembled data with 71.6% GC content. The N50 contig size was 6,580,622, and the genome contained 7131 protein-coding sequences (Figure 3) containing 84 pseudogenes, 18 rRNAs, and 94 tRNAs. Table 2 shows the details of the functional annotation of the genome. The obtained sequences were deposited at GenBank with accession number CP086119.

### 3.5. Genetic Basis for Secondary Metabolites

**Figure 4:**
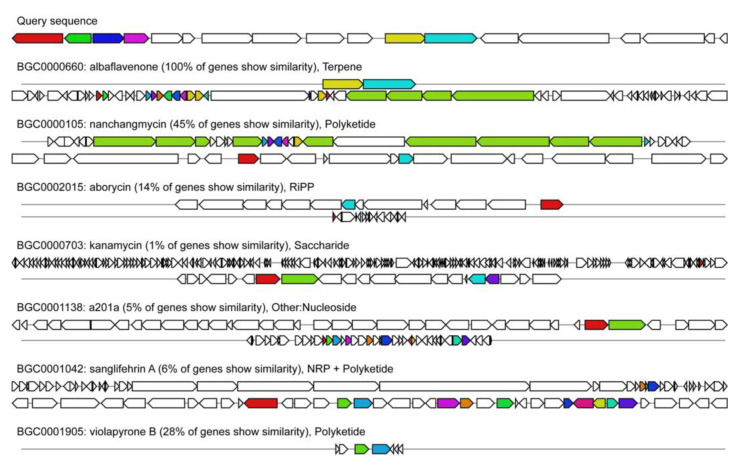
The similar BGC clusters detected for genes encoding compounds with antimicrobial activity predicted using anti-SMASH v. 6.0.1.

Anti-SMASH can predict different types of BGCs in the bacterial genome that encode potential secondary metabolites. The anti-SMASH analysis of *S. huasconensis* D23 showed the presence of 33 potential secondary metabolites BGCs (Table S1). One PKS-I and two PKS-III, eight gene clusters containing PKS-1, two NRPS, five hybrid PKS/NRPS, one thiopeptide/LAP, and three bacteriocins were found. Various types of BGCs, such as one siderophore, one arylpolyene, two butyrolactones, two lasso peptides, and one class-i lanthipeptide, were also found. Figure 4 shows the cluster map of genes predicted to have antimicrobial activity using anti-SMASH v. 6.0.1. Among all putative biosynthetic gene clusters for antibacterial compounds, the similarity of albaflavenone was the highest, and the similarities of Nanchangmycin and violapyrone B were less than 0.5. In addition, the similarities of several aborycin, sanglifehrin A, A201a, and kanamycin were less than 0.1. We hypothesize that clusters of genes encoding such low similarity to known compounds may give rise to new naturally occurring bacterial inhibitory active products, the exact products of which are to be further investigated. In addition, ectoine, geosmin, and hopene clusters are frequently found in *Streptomyces* strains [31,32], and Ectoine usually provides cellular protection against osmolarity and acts as a multifunctional nutrient [33].

### 3.6. Analysis of the Predicted Proteins

**Figure 5:**
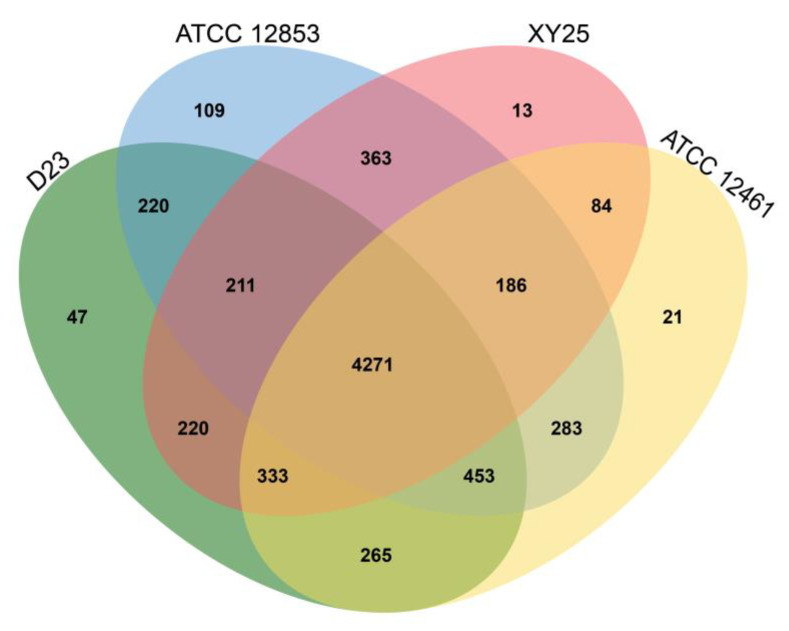
Venn diagram of comparative homologous protein analysis of the *S. huasconensis* D23, *S. alfalfae* XY25, *S. alboniger* ATCC12461, and *S. kanamyceticus* ATCC12853 genomes.

Comparative analysis of the genomes of *S. huasconensis* D23, *S. alfalfae* XY25, *S. alboniger* ATCC12461, and *S. kanamyceticus* ATCC12853 for homology-predicted protein-coding revealed that genes had significant gene overlap among the four strains. At the protein sequence level, analysis with OrthoVenn2 revealed 6993 clusters, 3516 orthologous clusters (at least containing two species), and 3477 single-copy gene clusters. There were 3621 homologous clusters common to all four strains; the number of homologous clusters shared by the three *Streptomyces* strains was 1707, with at least two genomes sharing 1399 clusters. A total of 266 gene clusters targeted only one genome, 53 of which belonged to the strain *S. huasconensis* D23 isolated in this study (Figure 5).

### 3.7. Cluster of Orthologous Groups (COG) Annotation

**Figure 6:**
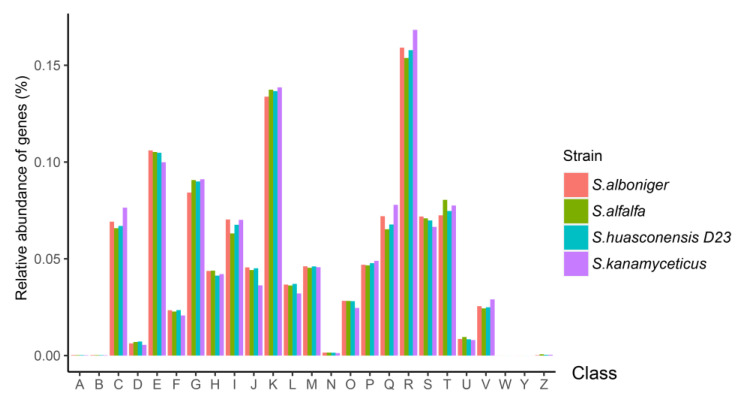
The cluster of Orthologous Groups (COG) database annotation of strain D23. The relative abundance of proteins (%) in the genome is shown. Letter codes are described in Table S2.

A total of 1848 genes were assigned to the COG databases for *S. huasconensis* D23. The numbers of genes annotated by COG were similar in the four strains (Figure 6, letter codes are described in Table S2); the genes that encode transcription accounted for the largest proportion of total genes in *S. huasconensis* D23 (13.7%). The genes that encode amino acid transport and metabolism accounted for 10.5% of *S. huasconensis* D23. Signal transduction mechanisms accounted for 8.04% of *S. huasconensis* D23. The most extensive features were attributed to the amino acids and their derivatives, followed by carbohydrate metabolism proteins, protein metabolism, fatty acids, lipids, isoprenoids and cofactors, vitamins, prosthetic groups, and cell wall and capsule and stress response for *S. huasconensis* D23 (Table S3). The KEGG metabolic pathway annotation results also showed that the number of genes involved in metabolism accounted for the vast majority (Figure S2). These annotations indicated the ability of *S. huasconensis* D23 to use the carbohydrates, amino acids, and protein resources available in their living environment.

### 3.8. Adaptation to Environment and Stress Responses

Microorganisms in the desert remain are exposed to harsh drought, extreme heat, and intense radiation, and they must be extremely well adapted to their environment in order to survive in the desert. The stress response in *S. huasconensis* D23 was investigated using SEED viewer version 2.0 [34]. The RAST annotation showed many subcategory distributions, and most features confirmed the COG analysis (Figure S1). From the subsystem classification, 462 genes were found to be associated with carbohydrate metabolism, of which 68 were aromatic compound metabolism-related genes. Next, 63 genes related to iron acquisition and metabolism, 11 genes related to dormancy and spore production, and 181 genes in response to environmental stress were also identified, which may play a crucial role in the survival of this strain in desert areas with high aridity and temperature differences. Overall, the functions associated with stress response in the *S. huasconensis* D23 genome include osmotic, oxidative, cold/heat shock, and perimetric stress responses (Table S3).

### 3.9. Core genome and Pan-Genome of S. huasconensis D23

**Figure 7:**
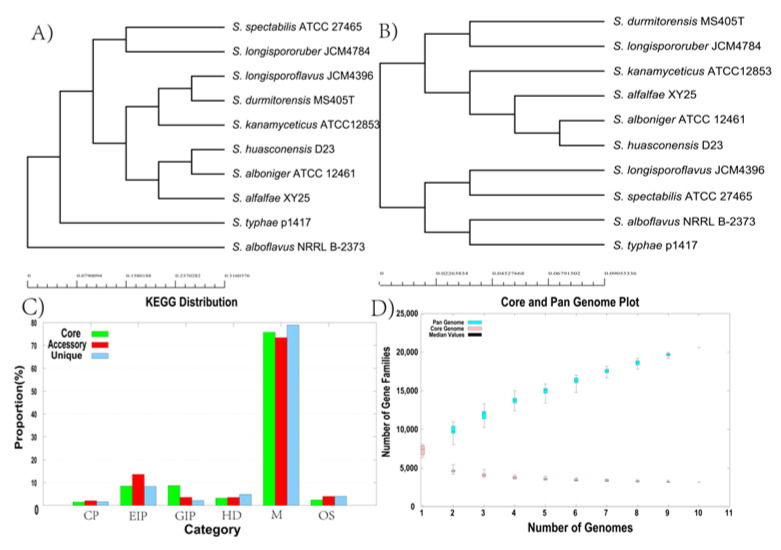
Pan-genomic analysis of *S. huasconensis* D23 and its evolutionarily related strains. (**A**) Neighbor-joining tree derived from 3165 pan orthologous proteins. (**B**) Neighbor-joining tree derived from core orthologous proteins. (**C**) KEGG analysis of 10 *Streptomyces* using core, auxiliary, and unique genes generated by pan-genomic analysis. Abbreviations: CP, Cellular Processes; EIP, Environmental Information Processing; GIP, Genetic Information Processing; HD, Human Diseases; M, Metabolism; OS, Organismal Systems. (**D**) Mathematical modeling of the pan-genome and core genome of strain D23 and its evolutionarily related strains.

By determining the core genome (number of genes common to all strains) and the pan-genome (number of core gene, auxiliary, and strain-specific genes) of *S. huasconensis* D23 and similar strains (Figure 7), we obtained information on the genomic variation and plasticity of the strain. For the 10 *Streptomyces* genome sequences included in the study, the size of the core genome decreased with increasing size of the pan-genome. The bar chart represents the distribution of core, accessory, and unique genes in several major KEGG categories. The largest number of genes is involved in metabolism, followed by environmental information processing, genetic information processing, human disease, organismal systems, and cellular processes (Figure 7C). Based on the correlation between the total number of genes and the number of genomes, the following fitted curve power-fit Curve equation can be elucidated: f(x) = 7215.01**x**^0.45^. The curve parameters indicate that the genome of D23 remains open (Figure 7D), containing 778 unique genes. In addition, the open pan-genome indicates a high potential for discovery of new genes as the number of strains increases.

## 4. Discussion

*Streptomyces* is a Gram-positive bacterium with high GC content that encodes broad smBGCs. The genome of *Streptomyces* typically possesses 25–70 BGCs producing over 10,000 bioactive compounds [9], such as antibiotics, anticancer drugs, antifungal drugs, and herbicides [35,36]. In recent years, an increasing number of studies have explored new sources of actinomycetes in extreme environments. Deserts characterized by high aridity, sparse vegetation, and strong radiation have been the treasure trove for the discovery of novel actinomycetes with unique genes and functions. Examples such as *Streptomyces sannurensis* sp. nov. [37], *Streptomyces xinjiangensis* sp. nov [38], and *Streptomyces altiplanensis* sp. nov. [39] were isolated from Wadi Sannur, Egypt; Lop Nur, Egypt; and Altiplano, Chile, respectively. Previous studies isolated a lasso peptide from the fermentation product of *S. huasconensis* [18] and found that its crude extracts exhibited 100% growth inhibition against *Propionibacterium acnes*, *Xanthomonas campestris*, NIH-3T3 (mouse fibroblasts), and HepG2 (*hepatocellular carcinoma*). In addition, over 90% growth inhibition against *Staphylococcus epidermidis*, *MRSA* (methicillin-resistant *Staphylococcus aureus*), and *Septoria tritici* were found [40]. We included more pathogenic bacteria for testing and found that the secondary metabolites of *S. huasconensis* also showed considerable growth inhibition activity against the newly included bacterial indicator strains. Our study expands *S. huasconensis’* spectrum of bacterial growth inhibition by synthesizing secondary metabolites.

Genome mining and metabolite analysis indicate that the isolated strains have a great potential for secondary metabolite production. Four BGCs encode known natural products: Ectoine (cluster 8, ectoine), albaflavenone (cluster 15, terpene), geosmin (cluster 24, geosmin), and citrulassin D (cluster 30, lassopeptide), Albaflavenone with a zizaene skeleton was isolated from a morphologically novel, highly odorous *Streptomyces* species in 1994 [41]. Albaflavenone, a tricyclic sesquiterpenoid antibiotic, is biosynthesized in *Streptomyces* coelicolor A3(2) by an enzyme encoded by a double-gene operon [42]. Recent studies have found that two new Albaflavenones isolated from Dictyophora indusiata inhibit the secretion of TNF-α and NO to varying degrees, showing anti-inflammatory activity [43]. Such a high degree of similarity leads us to speculate that the inhibition observed in our experiments is related to this compound. In addition, five gene clusters were more than 50% similar to known clusters. Six BGCs were identified in the genome of strain D23, for which the encoded compounds remain to be determined. Among the predicted results, we found seven compounds with bacteriostatic activity. The polyether antibiotic Namchamycin has a similarity of 0.45 and usually inhibits the growth of Gram-positive bacteria, such as *Mycobacterium*, and fungi affecting cation transport in the mitochondria [44,45]. The cluster 1-Aborycin similarity was 0.14. It was first isolated from *Streptomyces griseoflavus*, a tricyclic 21-peptide antibiotic, and earlier studies found that it has anti-infective activity, with moderate antibacterial activity against *Staphylococcus aureus*, *Enterococcus faecalwas* ATCC 29212, and *Bacillus thuringienswas* [46]. The similarity of cluster 3 to a polyketide violapyrone B with anti-influenza virus activity is 0.28 [47]. A201A is a structurally specific nucleoside antibiotic that was isolated from the metabolite of *Streptomyces capreolus* by Kirst [48]. It shows strong antibacterial activity against Gram-positive bacteria and most anaerobic Gram-negative bacteria [49,50]. However, the similarity of A201A is only 0.05. In addition, sanglifehrin A has a similarity of only 0.06, and kanamycin has a similarity of 0.01. Sanglifehrin A (SFA), isolated from *Streptomyces flaveolus* DSM 9954, a macrolide antibiotic, was shown to be a novel immunosuppressive agent in vitro experiments [51]. SFA has inhibitory effects against HCV/HIV [52]. Kanamycin (Km) is an aminoglycoside antibiotic produced by *Streptomyces kanamyceticus* [53] and has been used clinically since it was isolated in 1957 [54]. For the antibacterial activity, we observed secondary metabolite gene clusters, in *S. huasconenswas* D23 fermentation broth which were similar to known highly active compounds and may have played a critical role.

Desert microbes are subjected to periodic nutrient deprivation and various environmental stresses, and these abiotic and biotic stresses can pose a serious threat to survival. Bacteria must respond and adapt in this environment in order to survive. Due to several characteristics, including sporulation, wide metabolic capacity, competitive advantages via secondary metabolite synthesis, and multiple UV repair mechanisms, Actinobacteria is the dominant phylum in arid environments [55,56,57,58,59]. It has been shown that desert environments are rich in radiation-resistant strains of bacteria [21], among which there are some strains of Actinobacteria with outstanding anti-ionizing radiation activity [60,61]. Desert microbes respond to drought and extreme temperature stress by increasing the abundance of genes involved in osmoregulation and dormancy, and the increase in these genes can help them survive in such a harsh environment [62]. In the study of *Streptomyces avermitilis*, heat shock protein (HspR) was found to be important for the molecular mechanisms of growth and development, antibiotic production, and peroxide stress response [63]. The abundance of temperature-adapted genes (Table S3) in *S. huasconenswas* D23 may also be important for its good antibiotic production capacity and tolerance to peroxide stress.

Annotation of the strain D23 genome in subsystems revealed that most genes were associated with amino acid metabolism and derivatives (19.2%), closely followed by carbohydrate metabolism (12.9%), fatty acids, lipids, and isoprenoids (8.5%). These factors have an important role in the genetic regulation of the cellular response to the external environment and, in general, they protect the cell by participating in the regulation of gene expression. Among them, both oxidative stress and protection from reactive oxygen species can resist oxidative damage to cells caused by high radiation in desert environments, which may play an important role in the strain’s survival in harsh environments. Ectoine is a substance widely found in the intracellular compartments of bacteria and archaea, and it plays a protective role for cells under stressful external conditions [64]. For example, the presence of Ectoine can mitigate UV light, which damages cells under neutral conditions [65]. In addition, it is widely used in the cosmetic and pharmaceutical industries due to its moisturizing and anti-UV properties [66]. Geosmin is a class of intracellular organics that is widely present in actinomycetes and can be released extracellularly when the actinomycetes are damaged by oxidation [67]. Hopene is a class of hopanoids. Hopanoids interact with glycolipids in the bacterial outer membrane to form a highly ordered bilayer in a manner similar to the interaction of sterols with sphingolipids in eukaryotic plasma membranes [68]. It has been shown that hopanoids are essential for growth at higher temperatures, membrane permeability, and tolerance to low divalent cation concentrations [69]. Cold shock to the CspA family of proteins [70] and heat shock to the DnaK gene cluster extended may play key roles in bacterial adaptation to extreme temperature differences in deserts. Sigma B (σ B) plays a role in resistance to a variety of stressors, such as high and low pH, heat, high osmolarity, high ethanol concentrations, and oxidizing agents [71,72,73]. The stress responses investigated in this study identified the presence of BGCs in strain D23, which could be involved in adaptation to the stress caused by the desert environment.

## 5. Conclusions

In this study, we tested strain D23, isolated from a desert environment for its antibacterial activity, UV-C radiation resistance, and antioxidant capacity. In addition, we explored the molecular basis of the strain’s ecological adaptation at the whole-genome level. Strain D23 showed the highest recorded antibacterial activity against *Bacillus cereus*. The fermentation effect of using high nutrient medium Gauze’s Synthetic Medium NO. 1 was the best among the three mediums. Strain D23 can withstand UV-C irradiation up to 100 J/m^2^ (D10 = 80 J/m^2^) and tolerate stress up to 70 mM H_2_O_2_. Genome sequencing obtained an 8.2 Mb linear chromosome and predicted 33 secondary metabolite biosynthetic genes. Abundant and diverse secondary metabolite production capacity and plentiful stress-responsive genes ensure it can survive in extreme ecological niches. At the protein sequence level, analysis with OrthoVenn2 revealed significant gene overlap among strain D23 and closed evolutionary relationship strains. The pan-genome analysis of 10 strains of *Streptomyces*, including this isolate, revealed that the genome of D23 remained open. This implies that the possibility of discovering new genes is high as the number of strains increases. COG annotation found the largest proportion of genes encoding transcripts in the genome of strain D23. From the subsystem classification, 462 genes were found to be associated with carbohydrate metabolism. Stress-related functions predicted in the subsystem classification of the strain D23 genome include osmotic, oxidative, cold/heat shock, and peripheral stress responses. The predicted research opens the way for more in-depth studies of microbial adaptation and genomic evolution in desert environments. The genomic sequence data and smBGCs information are valuable for researchers interested in isolating bioactive compounds and working on the heterologous expression of cryptic BGCs for novel bioactive compound production.

## Figures and Tables

**Table 1:** Diameter of the clear zone (mm) of crude fermented extract of *S. huasconensis* D23.

	*B*.c	*B*.th	*S*.a	*E*.c	*P*.p	*L*.l	*M*.l	*S*.ep
CASM	30 ± 3	15 ± 9	25 ± 1	14 ± 2	10 ± 9	-	-	-
ISP2	9 ± 4	-	-	-	-	9 ± 1	12 ± 4	-
R2A	22 ± 4	-	-	-	-	-	-	19 ± 1

**Table 2:** General genome feature of *S. huasconensis* D23, *S. alfalfae* XY25, *S. alboniger* ATCC12461, and *S. kanamyceticus* ATCC12853.

Characteristics	D23	*S. alfalfae* XY25	*S. alboniger* ATCC12461	*S. kanamyceticus* ATCC12853
Size (bp)	8,236,582	8,273,342	7,962,786	10,133,897
GC Content (%)	71.6	72.2	71.2	71.0
Number of Contigs (with PEGs)	1	21	1	1
Number of Coding Sequences	7131	7175	6840	8762
Number of tRNA	94	85	81	80
Number of rRNA	18	17	18	18

## Data Availability

All data generated or analyzed during this study are included in this published article and its Supplementary Materials. The genomic sequences described here have been submitted to NCBI GenBank under the accession numbers OL824812 and CP086119.

## Supplementary Materials

The following supporting information can be downloaded at: https://www.mdpi.com/article/10.3390/microorganisms10122408/s1, Figure S1: A statistical overview of the genome coverage and annotated genomic subsystems of strain D23; Figure S2. Annotation results of KEGG metabolic pathway in strain D23; Table S1: Clusters of secondary metabolite genes encoded by *Streptomyces huasconensis* D23 predicted by anti-SMASH; Table S2: Cluster of Orthologous Groups functional classes. Abbreviations for Figure 5; Table S3: Stress response of *Streptomyces huasconensis* D23, using the SEED Viewer version 2.0.
