# Supplementary material for: Genomic Investigation of Desert Streptomyces huasconensis D23 Reveals Its Environmental Adaptability and Antimicrobial Activity
Source: Microorganisms. 2022 Dec 5;10(12):2408. doi: 10.3390/microorganisms10122408 (PMC9784485; doi:10.3390/microorganisms10122408)
Supplement: Supplementary file 1 [file microorganisms-10-02408-s001.zip › supplementary/Additional file 1-D23-genomic investigation.docx]

**Additional file 1**


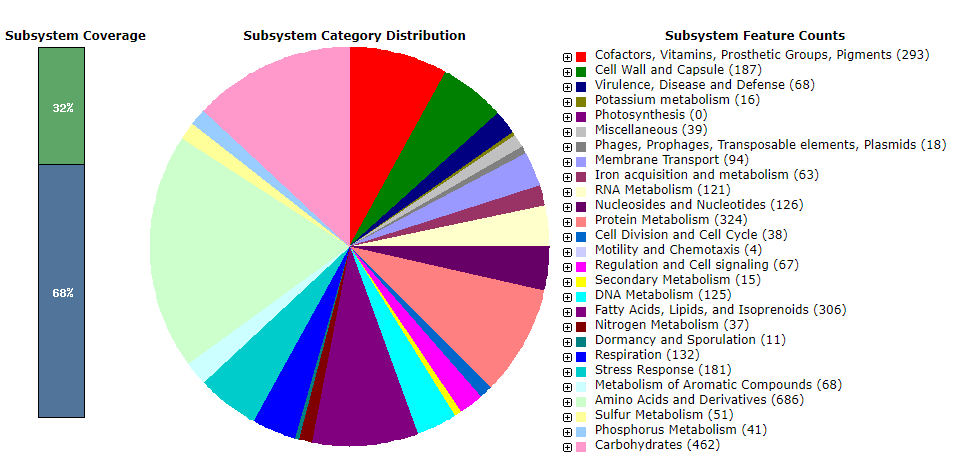


**Figure S1**. A statistical overview of the genome coverage and annotated genomic subsystems of strain D23


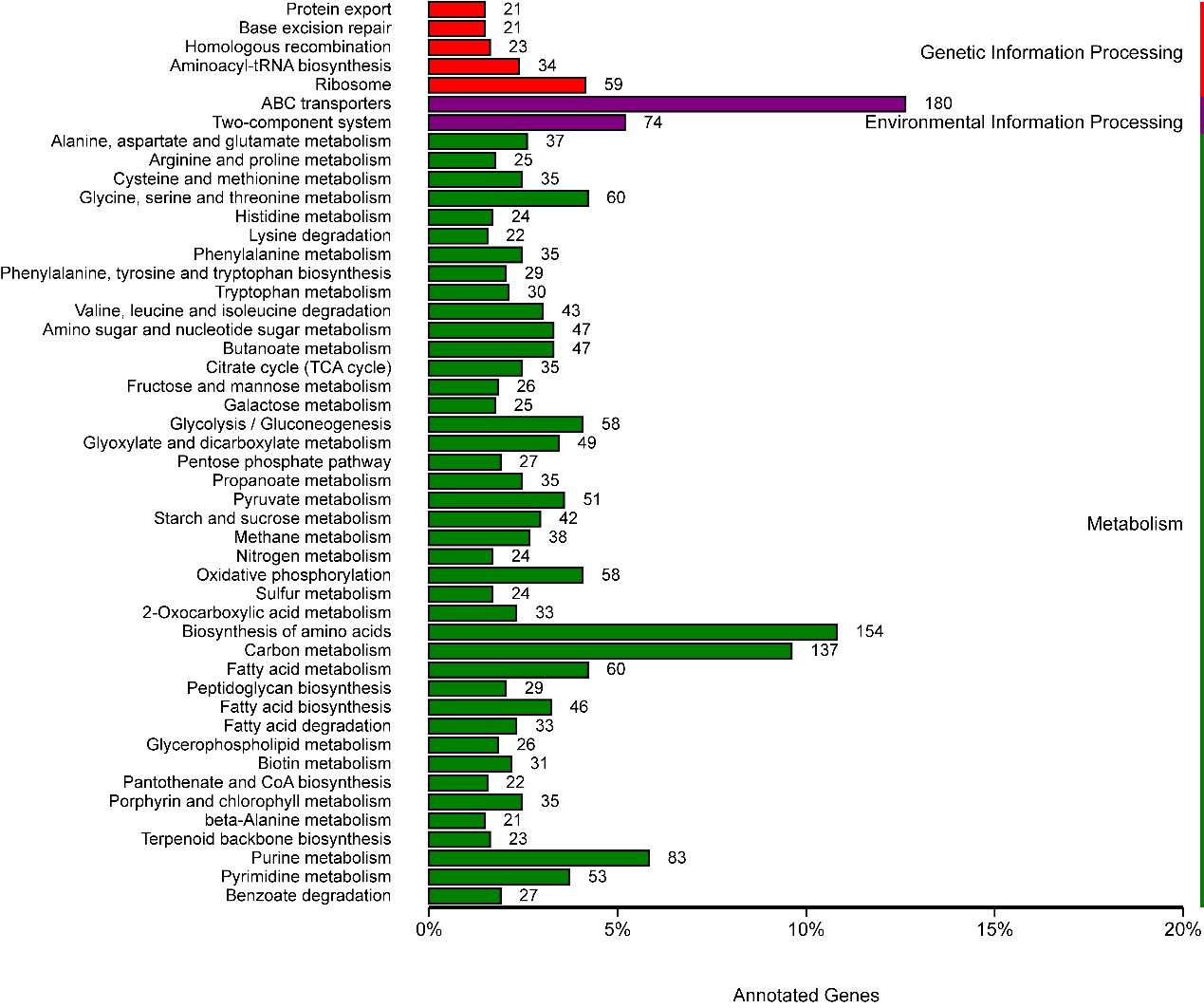


**Figure S2**. Annotation results of KEGG metabolic pathway in strain D23.
